# The British Journals

**Published:** 1841-10

**Authors:** 


### III. THE BRITISH JOURNALS.

*Memoir of the Case of a Gentleman born Blind, and successfully operated upon in the eighteenth year of his age; with Physiological Observations and Experiments.* By J. C. August Franz, M.D. M.R.C.S.

The young gentleman who is the subject of this memoir had been affected from birth with strabismus of both eyes; the right eye was amaurotic, and the left deprived of sight by the opacity both of the crystalline lens and of its capsule. At the age of seventeen, an operation for the removal of the cataract of the left eye was performed by the author with complete success. On opening the eye for the first time, on the third day of the operation, the patient described his visual perception as being that of an extensive field of light, in which every thing appeared dull, confused, and in motion, and in which no object was distinguishable. On repeating the experiment two days afterwards, he described what he saw as a number of opaque watery spheres, which moved with the movements of the eye, but when the eye was at rest remained stationary, and their margins partially covering one another. Two days after this the same phenomena were observed, but the spheres were less opaque and somewhat transparent; their movements were more steady, and they appeared to cover each other more than before. He was now for the first time capable, as he said, of looking through these spheres, and of perceiving a difference, but merely a difference, in the surrounding objects. The appearance of spheres diminished daily; they became smaller, clearer, and more pellucid, allowed objects to be seen more distinctly, and disappeared entirely after two weeks. As soon as the sensibility of the retina had so far diminished as to allow the patient to view objects deliberately without pain, ribands differently coloured were presented to his eye. These different colours he could recognize, with the exception of yellow and green, which he frequently confounded when apart, but could distinguish when both were before him at the same time. Of all colours, gray produced the most grateful sensation: red, orange, and yellow, though they excited pain, were not in themselves disagreeable; while the effect of violet and of brown was exactly the reverse, being very disagreeable, though not painful. Brown he called an ugly colour: black produced subjective colours; and white gave rise to a profusion of *muscæ volitantes.* When geometrical figures of different kinds were offered to his view, he succeeded in pointing them out correctly, although he never moved his hand directly and decidedly, but always as if feeling with the greatest caution. When a cube and a sphere were presented to him, after examining these bodies with great attention, he said that he saw a quadrangular and a circular figure, and after further consideration described the one as being a square, and the other a disc, but confessed that he had not been able to form these ideas until he perceived a sensation of what he saw in the points of his fingers, as if he really touched the objects. Subsequent experiments showed that he could not discriminate a solid body from a plane surface of similar shape; thus a pyramid placed before him, with one of its sides towards his eye, appeared as a plane triangle.

Two months after the above-mentioned operation, another was performed on both eyes, for the cure of the congenital strabismus, by the divisions of the tendons of the recti interni muscles, which produced a very beneficial effect on the vision of the left eye; and even the right eye, which had been amaurotic, gained some power of the perceiving light, and, from being atrophied, became more prominent. Still it was only by slow degrees that the power of recognizing the true forms, magnitudes, and situations of external objects was acquired. In course of time, the eye gained greater power of converging the rays of light, as was shown by the continually increasing capacity of distinct vision by the aid of spectacles of given powers.

*Proceedings of the Royal Society.* 1840-1841. No. 46.

---

*On the Preparation of Vinum Ferri.* By M. Donovan, Esq.

The virtues of this preparation, the first metallic medicine on record, have been known to mankind since the time of the Argonautic expedition: its medicinal character has therefore stood the test of experience during 3100 years—and it is now more in use than it has been for a long period. The will of the profession retains it, although it is expunged from the three British Pharmacopœias; and a medicine that has withstood such vicissitudes cannot be destitute of virtue. Why, then, is it expunged? why is there so much difference of opinion? The answer is, because there are such differences in its strength, and in the modes of preparation.

I have, by a very simple process, removed all uncertainty from this preparation, and now obtain it always of the same strength, containing an effective quantity of iron, of an agreeable flavour, and still possessing all the aroma of the wine. The process is this:—Take of the best hock one pint; common rust of iron of the shops well levigated, two ounces. Introduce both into a matrass, which plunge into a water bath maintained at the temperature of 100°. Constantly agitate the matrass for an hour; then remove it from the water, and the next day filter. The colour of this vinum ferri is a very deep greenish brown, almost black when the volume is great: its taste is ferruginous, agreeably and highly vinous; it produces a pleasant warmth in the stomach, and never sickens. In its effects it must be tonic, diuretic, emmenagogue, anthelmintic, and carminative. It does not, in a moderate dose, excite. No other wine than hock will afford a preparation possessing these virtues. The dose for an adult may be three or four drachms thrice a day; in smaller doses, it is of little use. If it is to be exhibited in combination with a bitter, it agrees well with colombo or gentian. By this method, in one day, we obtain a far better preparation than is procurable by the processes of the pharmacopœias in two months. The iron exists in it chiefly in the state of protoxide.

*Dublin Medical Press. June* 23, 1841.

---

*Hints to Prescribers of Sulphate of Quinina.* By M. DONOVAN, ESQ.

THE agreeable taste, odoriferous smell, and elegant colour of the acid infusion of roses have rendered it a favorite vehicle for the exhibition of more active medicines; and the choice is the more fortunate when the medicinal or physical properties of the infusion assist those of the remedy for which it is the selected medium. On these accounts the infusion of roses is frequently employed as a solvent for sulphate of quinina. The latter being nearly insoluble in water, and while in the insoluble state being much less active, it is rarely prescribed in liquids merely aqueous: sulphuric acid is therefore always employed to acidulate the water, as well as to add to the tonic powers of the salt. Infusion of roses contains the necessary quantity of sulphuric acid, and if it possess any medical efficacy, this coincides with the powers of the sulphate of quinina. Hence it is a common practice to prescribe infusion of roses with sulphate of quinina; and it is supposed that the two medicines form an elegant, efficacious, and compatible mixture.

I believe that the supposition is ill-founded. The mixture is not elegant; for it is no longer red and transparent, but becomes muddy and disagreeable in appearance; it is not efficacious; for much of the quinina is withdrawn in an insoluble state; and it is not compatible; for there are two sources of decomposition. Rose-leaves contain both gallic and tannic acids: hence gallate and tannate of quinina will be formed; both are insoluble in cold water; and they will remain floating through the liquid, notwithstanding the presence of sulphuric acid, which, so far as these salts are concerned, affects no good purpose as it does not dissolve the new salts formed.

Sulphate of quinina is also prescribed in conjunction with compound infusion of orange-peel as a vehicle and adjuvant. I have seen this much practised both in England and Ireland, but believe the formula to be liable to the same objection, for a precipitate is copiously deposited.

It might be supposed that it is little matter in what state the quinina is administered, whether as a sulphate, gallate, or tannate: but if the sulphate require the addition of sulphuric acid to hold it in solution, and if the state of solution be necessary to the exertion of its full medical efficacy, it must be improper to conjoin with it any agent which eliminates it in the solid form. Besides, it has never been proved that either the gallate or tannate of quinina possesses medical powers.

It seems much better to prescribe the sulphate of quinina simply dissolved in water, or in camphorated mixture, by the aid of a little sulphuric acid. If adjuvants are required, they may be administered by themselves, in some time after the exhibition of the sulphate. It is unsafe to combine or modify such delicate articles, as it is difficult to foresee where injury may be done. Who, except one that had witnessed the effect, would suppose that there could be any impropriety in conjoining sulphate of quinina with preparations of cinchona bark? yet alcoholic solution of sulphate of quinina is copiously precipitated by tincture of bark, and insoluble tannate of quinina is soon separated. I have reason to believe that the precipitate which falls when the decoction of bark is allowed to cool, consists chiefly of tannate of quinina.

*Dublin Medical Press. July,* 1841.

---

*On the Chorda Dorsalis.* By MARTIN BARRY, M.D. F.R.S. L. & E.

THE author of this communication, after pointing out the similarity in appearance between an object noticed by him in the mammiferous ovum, and the incipient chorda dorsalis described by preceding observers in the ova of other vertebrata, mentions some essential differences between his own observations and those of others as to the nature and mode of origin of these objects, and their relation to surrounding parts. Von Baer, the discoverer of the chorda dorsalis, describes this structure as "the axis around which the first parts of the fœtus form." Reichert supposes it to be that embryonic structure which serves as "a support and stay" for parts developed in two halves. The author's observations induce him to believe that, instead of being "the axis *around which* the first parts of the fœtus form," the incipient chorda is the last-formed row of cells, which have pushed previously-formed cells farther out, and that, instead of being merely "a support and stay" for parts developed in two halves, the incipient chorda occupies the centre out of which the "two halves" originally proceeded as a single structure, and is itself in the course of being enlarged by the continued origin of fresh substance in its most internal part.

The author enters into a minute comparison of the objects in question; from which it appears that the incipient chorda is not, as Baer supposed, developed into a globular form at the fore end, but that the linear part is a process from the globular; and that the pellucid cavity contained within the latter—a part of prime importance, being the main centre for the origin of new substance—is not mentioned by Von Baer. Farther, that the origin of the "laminæ dorsales" of this naturalist (the "central nervous system" of Reichert) is not simultaneous with, but anterior to, that of the chorda.

The author then reviews the observations of Rathke and Reichert on the chorda dorsalis, which contain internal evidence, he thinks, of a process in the development of fishes, reptiles, and birds, the same as that which he has observed in mammalia, namely, the origin of the embryo out of the nucleus of a cell.

And it is his opinion that this observation may assist to solve a question on which physiologists are not agreed; for it shows that if the nucleus of a cell is a single object, the first rudiments of the embryo are not two halves. The author thinks that unless the very earliest periods are investigated, it is in vain that we attempt to learn what that is of which the rudiments of the embryo are composed. From not attending to this, physiologists have supposed their "primitive trace" to arise in the substance of a membrane, which the author, in his second series on the embryo, showed could not be the case. To the same cause he thinks is referrible an opinion recently advanced by Reichert, that the first traces of the new being are derived from cells of the yelk.

*Proceedings of the Royal Society.* 1841. No. 46.

---

*On the Corpuscles of the Blood.* By MARTIN BARRY, M.D. F.R.SS. L. & E.

THE observations recorded in this memoir are founded on an examination of the blood in every class of vertebrated animals, in some of the invertebrata, and in the embryo of mammalia and birds. The nucleus of the blood-corpuscle, usually considered as a single object, is here represented as composed, in some instances, of two, three, or even many parts; these parts having a constant and determinate form. In the substance surrounding the nucleus, the author has frequently been able to discern, not merely "red colouring matter," but cell-like objects; and he points out an orifice as existing at certain periods in the delicate membrane by which this substance is surrounded. In a former memoir he had differed no less from previous observers regarding "cells." He had shown, for instance, that the nucleus of the cell, instead of being "cast off as useless and absorbed," is a centre for the origin, not only of the transitory contents of its own cell, but also of the two or three principal and last-formed cells, destined to succeed that cell; and that a separation of the nucleus into two or three parts is not, as Dr. Henle had supposed in the case of the pus and mucus-globule (the only instances in which the separation in question had been observed), the effect of acetic acid used in the examination; but that such separation is natural, apparently common to nuclei in general, and forming part of the process by which cells are reproduced. The author had farther shown the so-called nucleolus to be not a distinct object existing before the nucleus, but merely one of a series of appearances arising in succession, the one within the other, at a certain part of the nucleus, and continuing to arise even after the formation of the cell. These views he now confirms; and in the present paper shows that they admit of being extended to the corpuscles of the blood.

He then compares appearances observed in the latter with those he had traced in the ovum. These relate to the number of parts of which the nucleus is at different periods composed; the nature of the nucleolus; the communication between the nucleolus and the exterior of the cell; the formation of the contents of the cell out of the nucleus; the final division of the nucleus into the foundations of a limited number of young cells, destined to succeed the parent cell; and the escape of the young cells for this purpose. It follows from these investigations that the corpuscles of the blood are generated by a process essentially the same as that giving origin to those cells which are the immediate successors of the germinal vesicle, or original parent cell; it being also by a continuation of the same process that the corpuscle of the blood divides itself into the minuter objects figured by the author in his former paper on the blood.

He adds, that in its form and internal state the blood-corpuscle found in the adult of certain animals very much resembles that existing only in the fœtal life of others. It is incidentally remarked that the fœtal brain, at certain periods, appears to consist almost entirely of objects very much resembling those which, in some stages, form the nuclei in the fœtal corpuscles of the blood.

The author concludes by expressing his opinion that the mode of evolution of the minute mammiferous ovum is deserving of close attention, in connexion with some of the processes by which nourishment is communicated, and the growth of the body effected at all future periods of life.

*Proceedings of the Royal Society.* 1841. No. 46.

---

*On the Action of certain Inorganic Compounds when introduced directly into the Blood.* By JAMES BLAKE, Esq., M.R.C.S.

AFTER some preliminary remarks on the mode in which the experiments were conducted, and on the assistance derived from the hæmadynamometer of Poiseuille (or instrument for measuring the pressure of the blood circulating in the vessels), the author gives a list of the various saline substances of which he noted the effects when they were severally injected either into the venous or the arterial systems, arranged according to the nature of those effects. He finds, in general, that all the salts having the same base exert similar actions when introduced directly into the blood. He carefully enquires into the phenomena apparently arising from the direct contact of each of the substances above enumerated with the animal tissues; and more particularly into the effects produced on the heart, on the muscular and the nervous tissues, and on the pulmonary and systemic capillaries.

The first series of experiments related are those on the action of the salts of magnesia: these are found, when introduced in any quantities into the blood, to arrest altogether the action of the heart; but a still more remarkable effect which results is the complete prostration of muscular power. The salts of zinc have a similar operation under the same circumstances, but produce the same effects in smaller quantities. The action of the salts of copper, of lime, of strontia, of baryta, and of lead, are considered successively in the order in which they are more closely related by their physiological actions. The author particularly notices the peculiar action which the salts of the three last-named substances exercise on the muscular tissues, occasioning contractions in them during many minutes after death produced by their introduction into the blood. These muscular movements were, in some cases, observed forty-five minutes after the cessation of the heart's action. Experiments with the salts of silver and of soda are then detailed: substances which exhibit a remarkable similarity in their actions on the pulmonary tissue, on the heart, and on the systemic capillaries: for while, in the case of all the other salts already mentioned, death seems to be produced by the destruction of the irritability of the heart, the fatal result with the salts of silver and of soda is the consequence of their action on the tissue of the lungs. The physiological actions of the salts of ammonia and of potass were found by the author not to correspond with any of the preceding. Although agreeing perfectly with one another in their action upon the heart and systemic capillaries, they differ extremely in their effects on the nervous tissue; ammonia being particularly distinguished from all inorganic compounds in this respect, and being very analogous to poisons derived from organic products, which it also resembles in its chemical properties.

The general conclusion which the author is led to draw from these researches is, that there exists a close relation between the chemical properties of the substances experimented upon, and their physiological effects; his experiments tending to prove that, when introduced into the blood, substances which are isomorphous exert similar actions on the living tissues. He notices, however, two exceptions to this law; namely, the similarity of the actions exerted on the pulmonary tissue by the salts of lead with those of silver, although these salts are not isomorphous; and also the action on the nervous tissue of the salts of ammonia being different from that of the salts of potass. But he remarks that the oxide of lead bears a close analogy to the oxide of silver in its relation to organic compounds. The general fact previously announced by the author in his memoir read to the Academy of Sciences at Paris, namely, that salts with the same base have analogous actions, may be considered as a corollary of the above law.

*Proceedings of the Royal Society.* 1840—1841. No. 46.

---

*Researches tending to prove the Non-vascularity of certain Animal Tissues, and to demonstrate the peculiar uniform mode of their Organization and Nutrition.* By Joseph Toynbee, Esq. Member of the Royal College of Surgeons.

In the introduction to this paper, the author first speaks of the process of nutrition in the animal tissues which are pervaded by ramifications of blood-vessels; pointing out the circumstance, that even in them there is a considerable extent of tissue which is nourished without being in contact with blood-vessels. The knowledge of this fact leads us to the study of the process of nutrition in the non-vascular tissues; which tissues he divides into the three following classes; namely, first, those comprehending articular cartilage, and the cartilage of the different classes of fibro-cartilage. Under the second head he comprises the cornea, the crystalline lens, and the vitreous humour; and, under the third, he arranges the epidermoid appendages; viz. the epithelium, the epidermis, nails and claws, hoofs, hair and bristles, feathers, horn and teeth.

The author then proceeds to show that the due action of the organs, into the composition of which these tissues enter, is incompatible with their vascularity. In proof of the non-existence of blood-vessels in these tissues, he states that he has demonstrated, by means of injections, that the arteries, which previous anatomists had supposed to penetrate into their substance, either as serous vessels, or as red-blood vessels too minute for injection, actually terminate in veins before reaching them; he also shows that around these non-vascular tissues there are numerous vascular convolutions, large dilatations and intricate plexuses of blood-vessels, the object of which he believes to be to arrest the progress of the blood, and to allow a large quantity of it to circulate slowly around these tissues, so that its nutrient liquor may penetrate into and be diffused through them. The author states that all the non-vascular tissues have an analogous structure, and that they are composed of corpuscles, to which he is induced to ascribe the performance of the very important functions in the process of their nutrition, of circulating throughout, and perhaps of changing the nature of the nutrient fluid which is brought by blood-vessels to their circumference. The author then brings forward facts in proof of the active and vital properties of these corpuscles, and concludes his Introduction by stating, that it appears to him, that the only difference in the mode of nutrition between the vascular and the non-vascular tissues is, that in the former, the fluid which nourishes them is derived from the blood that circulates throughout the capillaries contained in their substance; whilst, in the latter, the nutrient fluid exudes into them from the large and dilated vessels that are distributed around them: and that in both classes, the particles of which the tissues are composed derive from this fluid the elements which nourish them.

The author then enters on an examination of the structure and mode of nutrition of the several tissues of each of these three classes. In considering the first class, he commences with articular cartilage, which he describes at great length in the various stages of its development, and at the different periods of life. He gives in detail the account of numerous dissections of the ovum and fœtus illustrating the first stage, during which he shows that no blood-vessels enter into the substance of any of the textures composing a joint; but that the changes its component parts undergo, are effected by the nutrient fluid from the large blood-vessels, by which, at this stage, each articulation is surrounded. In the second stage of the development of articular cartilage, the author shows, by numerous dissections, the process by which the blood-vessels are extended into the substance of the epiphysal cartilage, and converge towards the attached surface of articular cartilage, and how, at the same time, blood-vessels are equally prolonged over a certain portion of its free surface. He shows that none of these blood-vessels enter the substance of the articular cartilage, and he points out that in them the arteries become continuous with the veins; first, by their terminating in a single vessel, from which the veins arise; secondly, by their forming large dilatations from which the veins originate; and, lastly, they become directly continuous with the veins in the formation of loops of various characters. In the third stage, that which is exhibited in adult life, the epiphysal cartilage is converted into osseous cancelli. These contain large blood-vessels, which are separated from the articular cartilage by a layer of bone composed of corpuscles, and the author believes that the principal source of nutrition to this tissue is the nutrient fluid which exudes into it from these vessels, by passing through the articular lamella just noticed. The free surface of adult articular cartilage is nourished by vessels which pass to a slight extent over it. The author points out the presence of fine tubes which pervade the attached portion of adult articular cartilage, to which he ascribes the function of transmitting through its substance the nutritive fluid derived from the vessels of the cancelli. He also advances the opinion that the articular cartilage becomes thinner during the whole of life, by being gradually converted into bone.

Fibro-cartilage constitutes the second tissue of the first class. The author first enters upon an examination of its structure; and in order to arrive at some definite conclusions on this subject, whereon anatomists of all ages have so much differed, he made numerous dissections of fibro-cartilages in the different classes of animals at various periods of their development, the results of which he details. He arrives at the conclusion that this tissue is composed of cartilaginous corpuscles and of fibres; the latter preponderating in adult life, the former in infancy; and that during life the corpuscles are gradually converted into fibres. He enters at length into the question of the vascularity of these cartilages; and from a careful study of many injected specimens of man and animals at various periods of their development, the particular results of which he relates, he believes that blood-vessels are contained only in their fibrous portion, and have the function of nourishing that which is cartilaginous, and which, on account of its being subject to compression and concussion, does not contain any.

Among the second class of extra-vascular tissues, the cornea is first treated of; and its structure is described as being very lax, and as containing corpuscles only in a small quantity. The opinions in favour of its vascularity are combated; and it is shown that the blood-vessels which converge to its attached margin, and which are the principal source of the fluid that nourishes it, are large and numerous, and that at the circumference of this tissue the arteries, without any diminution of their caliber, return in their course, and become continuous with the veins. A second set of vessels, devoted to the nutrition of the cornea, is also described; they extend to a short distance over the surface of the tissue, but do not penetrate into its substance.

The crystalline lens is described as being composed of corpuscles, of which the radiating fibres are constituted. The arteria centralis retinæ is described as ramifying over the posterior surface of the capsule, where it forms large branches; these pass round the circumference of the lens, and reach its anterior surface, at the periphery of which they become straight: the arteries terminate in loops frequently dilated, and become continuous with the veins. With respect to the vascularity of the vitreous humour, the author states that although many anatomists have, in general terms, represented the arteria centralis retinæ as giving off, in its course through this organ, minute branches into its substance, still those who have paid especial attention to the subject, have not been able to find such vessels. He believes that the nutrition of this structure is accomplished by the fluid brought to its surface by the ciliary processes of the choroid, which fluid is diffused with facility through its entire substance by means of the corpuscles of which its membrane is composed, assisted by the semifluid character of the humour.

The third class of extra-vascular tissues comprehends the epidermoid appendages. The author describes them all as composed of corpuscles, which are round and soft where they are in contact with the vascular chorion, compressed and flattened where they are farther removed from it. He points out, in the substance of the hoof of the horse, the existence of fine canals, which he supposes to conduct fluid through its mass; and he states that the perspiratory ducts of the human subject possess a structure analogous to the spiral vessels of plants. The author describes each of the tissues of this class at length, and shows that the various modifications presented by the vascular system with which each is in contact, have the sole object of enabling a large quantity of blood to approach and circulate slowly around them. He also points out, in connexion with this subject, the remarkable vital properties which are possessed by these non-vascular tissues.

In concluding this paper, the author states that his object has been to establish as a law in animal physiology, that tissues are capable of being nourished, and of increasing in size, without the presence of blood-vessels within their substance. He shows the analogy which is presented between the extra-vascular animal and the extra-vascular vegetable tissues. He expresses a hope that the application to surgery of the above law, with reference to the prolongation of blood-vessels into the extra-vascular tissues during disease, and to pathology in the investigation of the nature of morbid structures, particularly of those classes which contain no blood-vessels, will be not devoid of interest, and will be productive of some advantage.

*Proceedings of the Royal Society.* 1841. No. 48.

